# An Address Delivered in Rush Medical College

**Published:** 1852-03

**Authors:** 


*An Address delivered in Rush Medical College, Nov. 3d,* 1851. By N. S. Davis, M. D., Prof. of Pathology, Practice, and Clinical Medicine. Published by the Class.

The subjects of this Address are "The dignity, honor, and welfare of the Medical Profession, on what do they depend?" The dignity, honor, and welfare of the medical profession, have occasioned of late not a little inkshed. It appears to be frequently taken for granted that in these three aspects the medical profession of the present time compares very unfavorably with the past; that it is far less honorable than in times gone by; that in dignity it has sadly degenerated, and that its welfare is less regarded. It would perhaps save some fruitless expenditure of rhetoric, and calm the ebullitions of an excited imagination, were it to be candidly considered whether, in these several points of view, the profession does not at the present moment hold, to say the least, as high a position as formerly — in other words, whether the premises upon which are based much of the rhodomontade which has become of late so common are not in reality falsely assumed.

The address by Prof. Davis contains some information respecting the state of the profession in this country at former epochs in its history. He says: "We are told on good authority, that in 1753, when New York city contained 10,000 inhabitants, among them were 'more than *forty* gentlemen of the Faculty, the greatest part of whom were mere pretenders to a profession, of which they were entirely ignorant.' 'In New England, the greater number of those who practiced Medicine were Priests, whose medical knowledge was chiefly derived from the writings of Hippocrates, Galen, Areteus, &c., which they had read during a collegiate course of education in Europe.'

"And even at a later period, during the first quarter of a century after the recognition of our National Independence, and after two or three Medical Schools had been established in the Atlantic cities, we are told by the late and eminent Dr. John Stearns, that 'those who witnessed the original and progressive settlement of the Northern and Western sections of New York State, since the year 1791, will recognize the mania that infatuated the emigrants from the East, and the ambitious projects formed by those who assumed the title of Doctor.

"Many who had never read a volume in Medicine, were suddenly introduced to an extensive practice, and to a reputation of such imposing authority, as to control the opinions of their superiors in science, and to prescribe rules of practice for their government. Consultations were generally characterized by gross controversies at the bed-side of the patient, whose health and life were often immolated to the ignorance, prejudices, or discordant theories of the contending Physicians. With few honorable exceptions in each county, the practitioners were ignorant, degraded, and contemptible."

The truth is, that, in every point of view, the history of the medical profession in this country up to the present time, will by no means show a decline and fall. That the knowledge of disease, and of the means of curing disease is far greater than at any past period, will probably not be doubted. The disposition to undervalue our own generation expends itself in what pertains to the profession, rather than to the science. Now we think it would not be difficult to prove, by historical facts and testimony, that the members at this moment constituting the medical profession in our country, as a body, are as honorable, as truly dignified, and as flourishing as at any former epoch. We have no hesitation in expressing the belief that in scientific attainments, American physicians and surgeons of the present day, as a whole, are not farther below the requisitions of science than has hitherto been the case.

This is far from saying that there is not abundant scope for raising the average of scientific knowledge and ability. The question is not whether a large portion of the medical profession are unlearned or unskillful, but whether the proportion of ignorance and mal-practice has not always been as great, or greater, than in our day. We have started a topic which we have no intention of pursuing at present. Prof. Davis does not discuss the question just raised. From the facts contained in the above citations, and others, he deduces *first*, the conclusion that "the *number* of physicians in any given community, does not bear any relation to, and is not in any wise dependent on, the means and facilities afforded for acquiring medical knowledge." A contrary opinion is sometimes assumed, as the ground of finding fault with the multiplication of medical schools. It is charged upon medical schools that they tend to overcrowd the profession. Prof. Davis, however, shows that "at a period in our history when there was not a medical college in America, and when a medical education could be obtained only by submitting to the trouble and expense of twice crossing the Atlantic, and spending several years in some one of the medical schools of Europe, New York city contained at least one doctor to every 250 inhabitants!" He adds, "I doubt whether any one will contend that she now contains a larger relative proportion, although containing in her midst *three* medical schools, and three more within the limits of the state."

We do not doubt Prof. D. to be correct in this view of the matter. In fact we believe that in proportion as the facilities for medical education are few and difficult, will the number of practitioners be increased rather than diminished, so long as the law does not interfere and forbid the practice of medicine except by those who have enjoyed certain prescribed opportunities. If the means of education are not within the reach of the majority of those who wish to become physicians, the result will be, not that there will be fewer physicians, but that the majority will be imperfectly educated. We think it might be shown that the most efficient mode of securing a higher grade of medical attainment, and at the same time restricting the undue multiplicatiou of practitioners, is to develope in the ranks of the profession a greater zeal for self-improvement. The character of the mass of those who are entering the profession is determined, in no small measure, by the character of those already in the profession, especially of senior members who enjoy the confidence of the community. Medical students at the time of graduation are constantly told that their studies are not ended — that they are to continue to be diligent in acquiring knowledge and keeping pace with the progress of science. To what extent is this important counsel enforced by the examples of those whom these young practitioners will likely to take as models for imitation? Medical Institutions undoubtedly tend to improve the character of the profession, each in the local circle over which its influence extends. In this way they contribute to raise the standard of medical acquirement, and, if properly conducted, the more numerous they are, the better, in this point of view. It is clear enough from reasoning and observation, that the citizens of a state in which public schools and academies abound, are more intelligent, as a body, than communities in which there is a lack of educational advantages. It would be singular inconsistency if the same rule did not apply to medical institutions.

A second inference deduced by Prof. D., is, "that the relative proportion of those in the profession who are well educated in all its departments, will depend directly on the facilities afforded for acquiring medical knowledge." With this inference the writer endeavors to sustain the policy adopted by the school with which he is connected, of lessening the fees for medical instruction to an amount nearly nominal. His arguments in fact, will apply to free medical instruction. The gist of the reasoning is this: The number of physicians is not lessened but increased by the difficulties (including expense) of obtaining a thorough medical education. Persons resolving to become physicians will avail themselves of the best facilities if these lie within their power, but if not, they will gain admission into the profession with inferior, or wholly without educational advantages. Hence, in proportion as the means of medical instruction are made available will they be enjoyed, and, as a consequence, the average of acquirement will be raised.

It must be admitted that there is considerable cogency in this train of argument. We are not prepared to dispute the correctness either of the premises or the deductions. The subject, however, is to be considered in other points of view. The objections to state medical instruction in this country, i. e., schools with teachers appointed and paid by government, as it appears to us, are insurmountable. It is enough that professorial chairs would, under this system, become part of the spoils of office, to be enjoyed by a dominant political party, and that bar-room politicians would be the successful candidates. Constituted as the colleges now are, to lower fees beyond a certain point, might perhaps reduce the competition to dollars and cents, irrespective of educational facilities. While emulation, as respects the latter, is no doubt serviceable to the cause of medical instruction, underbidding in price might be otherwise. Again, to command talent and effort, medical teaching must not be without some approach to pecuniary compensation. A person may lecture for a session or two for the mere honor, or as an incentive for personal improvement, but few will be willing to labor for many successive years without some reference to emolument. The facilities for medical instruction also involve pecuniary outlays for which some provision must be made in the resources of an institution. These considerations are to be taken into account in discussing the subject of cheapening medical education — a subject to which we may revert at some future time.

